# The Relationship Between Personality and Neurocognition Among the American Elderly: An Epidemiologic Study

**DOI:** 10.2174/1745017901713010233

**Published:** 2017-11-28

**Authors:** Nelson Mauro Maldonato, Raffaele Sperandeo, Silvia Dell'Orco, Pasquale Cozzolino, Maria Luigia Fusco, Vittoria Silviana Iorio, Daniela Albesi, Patrizia Marone, Nicole Nascivera, Pietro Cipresso

**Affiliations:** 1 Dipartimento di Neuroscienze e Scienze Riproduttive ed Odontostomatologiche, Università di Napoli Federico II, Napoli, Italy; 2 SiPGI Scuola di Specializzazione in Psicoterapia Gestaltica Integrata, Torre Annunziata, NA, Italy; 3 IRCCS Istituto Auxologico Italiano – Milano

**Keywords:** Personality, Neurocognitive function, Older adults, Big-five, Cognitive ability, Epidemiology

## Abstract

**Background:**

Although different personality traits have often been associated with different levels of mental activity and cognitive functioning, no previous studies have evaluated the association in a sample that mirrors a nationally-representative sample of elderly individuals.

**Objective:**

To evaluate the association between personality traits and neurocognitive functioning among individuals 51 years and older using the Cognition and Aging in the USA (CogUSA) database.

**Methods:**

We analyzed the association between personality traits and neurocognitive scores derived from Waves I and II of the study. Neurocognitive functions were modeled as an outcome variable using the Big Five Personality Traits as predictors.

**Results:**

All personality traits were associated with higher education except Conscientiousness. Older age was associated with higher levels of the Agreeableness and Openness traits. Extraversion, Conscientiousness and Openness were positively associated with increased neurocognitive function and self-rated present memory. Extraversion and Openness also had a positive association with long-term retrieval. Agreeableness was negatively associated with several neurocognitive functions, while Neuroticism was negatively associated with memory and cognitive effort.

**Conclusion:**

Extraversion, Conscientiousness and Openness personality traits are associated with good cognitive health. Individuals scoring high in Neuroticism and Agreeableness might benefit from tailored cognitive interventions to prevent age-related cognitive decline.

## 1 INTRODUCTION

Cognitive changes in the elderly can range from normal to pathological impairment, the latter affecting quality of life [1]. With the increasing life expectancy of the global population, there has been a proportional increment in the incidence of impaired neurocognition [2]. Maintaining high levels of neurocognitive functioning has been associated with the engagement in tasks that keep the elderly mentally active [3], this involvement is often being connected with previously existing personality traits [4]. Although different personality types have been associated with diverse levels of mental activity and neurocognitive functioning, these findings have usually been based on either local samples or multi-institutional prospective studies that are not necessarily representative of population health.

For a long time, behavioral scientists have examined how personality influences physical and mental health [5], some researchers suggest that personality may be related to neurocognitive functioning in the elderly [6, 7]. Personality traits have been classified through a wide range of scales, one approach being the Five Factor Model (FFM). This includes the 'Big Five' dimensions of personality, namely: Extraversion (people with high energy, sociable, and good at communication), agreeableness (having traits such as kindness, trust, and altruism), conscientiousness (including strong impulse control, focus on goals, reliability, and punctuality), neuroticism (being emotional, anxious, moody, and irritable), and openness to experience (imaginative insight with multiple interests) [8, 9]. These traits describe differences in typical cognitive and affective experiences, with implications for behavior (5). In addition, personality traits have been associated with altered levels of some brain neurotransmitters, affecting a range of behavioral activities [10]. For example, serotonin levels have been associated with neuroticism, agreeableness, and conscientiousness, while dopamine has been associated with exploratory behaviors [10, 11]. Personality may also affect the rate of cognitive decline through behavioral aspects including response to stress, health behaviors, and cognitively-stimulating activities [6, 12, 13]. For instance, individuals with strong tendencies toward negative emotions might undergo deleterious changes in cerebral structures over time [14, 15]. Conversely, positive traits such as openness and optimism may indirectly protect against cognitive decline by facilitating effective coping and life engagement [16]. A full understanding of the relationship between personality and cognitive functioning in the elderly is therefore necessary in developing interventions aimed at promoting a healthy cognitive aging.

A number of studies have explored the association between personality traits and neurocognitive functioning. For example, women with neuroticism -- specifically presenting signs of anxiety, jealousy and moodiness symptoms -- are associated with an increased risk of developing dementia and depressive symptoms in later life [17, 18]. Similarly, individuals with high scores for neuroticism and low scores for conscientiousness have demonstrated a threefold risk for developing Alzheimer's disease, while high scores for conscientiousness tend to reduce the risk of dementia [19, 20]. Common to all of these studies are the inferences from local samples with relatively smaller sizes, although it is questionable whether such inferences might apply to the overall population.

Faced with this gap in the literature, our objective was to evaluate the association between personality traits and neurocognitive functioning among individuals 51 years and older, through the COGUSA database, a sample that mirrors the main Health and Retirement Study sample [21]. We hypothesized that lower levels of Neuroticism and higher Openness, Extraversion and Conscientiousness would predict better cognitive function [22].

## 2 METHODS

This is a cross-sectional study to evaluate the association between personality types and neurocognitive functioning among individuals 51 years of age and older using the CogUSA database. We reported results following the STROBE (Strengthening the Reporting of Observational Studies in Epidemiology) Statement [23].

### 2.1 Ethics

Approval was sought and obtained from the Institutional Review Board at the University of Basilicata, Potenza, Italy.

### 2.2 Setting

Data were obtained from the Cognition and Aging in the USA (CogUSA) database, a longitudinal study collecting information on age-related cognitive changes and their impact on health, including individuals 51 years old and above. The CogUSA sample mirrors the main Health and Retirement Study sample [21], which is representative of the United States population. Data were collected in three waves: Wave I was a 40-minute telephone interview to obtain information on demography and neurocognitive tests, and lasted a week. Wave II was a three-hour face-to-face interview assessing neurocognitive function with the use of an extensive testing battery [24-27], the Need for Cognition Scale [28], and the Big Five Personality Traits tests [8]. Wave III was identical to Wave I, but conducted within one to 24 months following Wave II. In this study, we analyzed the association between personality traits and neurocognitive scores obtained in Waves I and II. Wave II was conducted within a week of Wave I, therefore our study is a cross-sectional analysis.

### 2.3 Participants

The CogUSA database contains information on 28 primary sample units in the United States, including participants born in 1956 or before. Investigators conducted a two-stage, random digit-dialing sampling method using information from the Genesys database (http://www.m-s-g.com/web/genesys/index.aspx, last accessed on November 2016]. The probability of selection from 28 primary sampling units determined the study sample weights, so that inferences could be made to the Health and Retirement Survey. Also, samples from Wave I were compared to the Health Retirement Survey 2004 sample, as both groups had participants with similar characteristics. A post-stratification was conducted using education, gender and rural/urban status. We excluded those who did not complete the cognitive interview and individuals who participated in both the CogUSA and the Health and Retirement Survey.

### 2.4 Outcome Variables

The CogUSA investigators evaluated neurocognitive function through the following tests and corresponding constructs during Wave I: Self-rated memory (1-5 scale, where 1 = excellent and 5 = poor memory), and self-rated past memory during the past two years rated as 1 = better; 2 = same; 3 = worse. Both tests showed good validity and reliability [29]. For wave II the following cognitive tests and corresponding constructs were applied:

1. The Wechsler Abbreviated Scale of Intelligence (WASI) full battery, assesses general intelligence and overall cognitive capabilities and consists of four sub-tests: a) Vocabulary - participants provide the four object names in pictures or defines 37 words presented to them. It measures semantic knowledge and verbal comprehension. b) Block design - participants complete a series of two-color pattern using blocks in limited given time and it measures spatial-visual ability and visual-motor coordination. c) Similarities - participants describe how similar are the two words or concepts and measures verbal concept formation and reasoning, and d) Matrix reasoning - participants view a matrix and select the correct response to complete the matrix. It measures inductive reasoning, non-verbal abstract problem solving and general intellectual ability [8]. Its reported validity and reliability provide adequate and empirical support for WASI tests [30, 31].
2. Woodcock Johnson Psychoeducational Test Battery (WJ-III) measures cognitive abilities and achievements in areas of reading, mathematics, written language and knowledge, and includes: a) Number series - respondent looks at the series of numbers with missing number, determines the pattern and identifies the missing number to complete a numerical sequence. It measures quantitative reasoning. b) Retrieval fluency - participant names as many words from the given categories in one minute, measuring long-term retrieval. c) Verbal analogies - participant completes the analogies with a correct phrase or word and measures reasoning ability using lexical knowledge. d) Spatial relations - respondent identifies the component parts to complete whole-shape measuring visual-spatial thinking. e) Picture vocabulary - respondent identifies familiar and unfamiliar pictured objects measuring their aspects of lexical knowledge. f) Auditory working memory - respondent listens to mixed series of words and digits and attempts to reorder them by first words in order and then the numbers in order. It measures the short-term working memory. g) Visual matching - participant quickly locates and circle two identical numbers in a row of six numbers within 3 minutes, measuring visual perceptual speed. h) Concept formation - participant identifies rule application and frequent rule-switching after being exposed to concepts, and measures inductive reasoning. i) Calculation - measures participant's ability to perform mathematical calculations including addition, subtraction, multiplication and division, and j) Word attack - participant reads non-words or low-frequency words aloud in English, and it assesses the skill in using phonic and structural analysis in pronouncing unfamiliar words [9, 26]. The psychometric properties of the WJ-III provides sufficient validity along with appropriate reliability when used in a variety of conditions [32-35]. Scoring for the Wechsler Abbreviated Scale of Intelligence full battery and Woodcock Johnson Psychoeducational Test Battery is done by adding up the raw scores (number correct, number of points, or number of errors) and is converted to age and grade equivalents, percentile ranks, and discrepancy scores with use of the scoring tables. The standard average score is 100 with higher scores representing better performance and lower scores, the worst performance (131 and above- very superior; 121 to 130 - superior; 111 to 120 - high average; 90 to 110 - average; 80 to 89 - low average; 70 to 79 - low; 69 and below - very low).
3. Need for cognition scale (NCS), defined as “the tendency for an individual to engage in and enjoy thinking,” is a validated scale measuring variables namely cognitive enjoyment and cognitive effort using a short 18-item form. The responses are calculated using a 5-point Likert scale ranging from strongly disagree to strongly agree [9, 28].
4. Extensive cognitive battery with sub-tests: a) Switching task, is a simple two-choice response task saying “stop” when respondent hears the word “red” and saying “go” to the word “green.” It measures speed processing by assessing attention, reaction time, task switching and inhibitory control [24]. The switching tasks showed high rates of accuracy, with good reliability and validation [24, 36, 37] and b) Vigilance task - consists of three trials: In the first one, participants are asked to locate a specific number each time it appeared from a row of numbers and report the number which came after this number in series. In the second one, the same task is repeated but with alphabets. The third trial includes both numbers and alphabets, where participants are asked to locate the number or alphabet after a specific number or after a specific alphabet. The task offers a reliable and valid measure of the attention and processing speed [25, 38]. Automatic scoring of verbal responses as well as measurement of response times is recorded by using speech recognition software and output into the excel spreadsheet.
5. Other cognitive measures involving episodic memory, a reliable and valid test which consists of immediate and delayed word recall and is scored as the total count of words recalled correctly. It involves acquisition, storage and retrieving information [29, 39].

### 2.5 Predicting Variables

The Big Five Traits Personality test classified participants into the following categories: 1) Extraversion, referring to sociability, assertiveness, energetic; 2) Agreeableness characterized by friendly, affectionate, altruistic, and trustworthy; 3) Conscientiousness including attributes such as highly organized, goal-oriented and disciplined; 4) Neuroticism including a tendency to worry, nervousness, and negative emotions and 5) Openness including characteristics such as being creative, inventive, and curious with a broad range of interests. All five traits are measured using the self-reported 44-item Big Five Inventory on a 5-point Likert scale (1 = strongly disagree to 5 = strongly agree). The scale is psychometrically valid and reliable with Cronbach's alpha reliability scores ranging from .75 to .90 and three-month test-retest reliability ranging from .80 to .90, with a mean of .85 [8].

### 2.6 Potential Confounding Variables

We selected potential confounders using a combination of clinical judgment and evidence from the literature, as these joint criteria have been demonstrated to perform better than the isolated selection of isolated clinical or evidence-based criteria [40]. Specifically, we selected educational level, age, race, gender, and marital status.

### 2.7 Data Analysis

Our exploratory analysis was started with a visual exploration of all variables to evaluate frequency, percentage and near-zero variance for categorical variables (Extraversion, Agreeableness, Conscientiousness, Neuroticism, and Openness personality traits), distribution for numeric variables (neurocognitive functions), and their corresponding missing value patterns [41]. Near zero variance is found when a categorical variable had a small percentage of a given category. Variable transformations, and dummy coding for variables with distributions that were not normal at inspection, variable re-categorization or removal for near-zero variation, and different imputation algorithms for variables with missing values [42].

Our modeling strategy made use of a series of generalized linear models with a Gaussian family, i.e., multiple linear regression models, to model the association between neurocognitive function and Big Five Personality Traits, adjusted for age, race, gender, educational level and marital status. In order to be able to calculate predicted means (Odds Ratio, OR) for the outcome rather than simply obtaining less clinically interpretable measures of correlation, we categorized personality traits using median values. Results are reported as predicted means (or medians for log transformed variables) with 95% confidence intervals, with results being interpreted as significant when the confidence intervals do not cross a value of 1.0.

All of our analyses were adjusted for the corresponding set of weights (multipliers relating the sample to the total population), strata (subpopulations) and primary sampling units (sample aggregates) since this dataset is representative of a larger population. These adjustments allow for our results to be inferred to the larger population rather than being applicable only to our study sample. In our study these inferences have two major implications. First, for each of our frequencies we report both the number of individuals in our study sample as well as in the corresponding overall population to whom these results apply. Second, our confidence intervals are adjusted to the population rather than to our study sample. In other words, our results represent the relationship between neurocognitive function and personality traits in the United States population.

## 3 RESULTS

In the initial analysis, we present an overall study sample description along with a stratification by personality traits. Personality traits are split in relation to their median values so that additional calculations in this manuscript can be used for population estimates. Our results present estimates to the target US population, which were calculated based on adjustments for survey characteristics. Frequencies in the tables represent inferences for the target population after adjustment for survey weights, strata, and primary sample units. The mean age of the population across all personality trait groups was 65 years with the majority represented by women (55.2% ± 4%). Most participants were white (90.8% ± 4.9%) and married (64.7% ± 4.2%), with more than 90% having completed high school in each group. All personality traits were associated with higher education except Conscientiousness. Older age was associated with higher levels of Agreeableness and Openness traits (Table **1**).

To compare the association between median personality trait levels and neurocognitive traits, we estimated predicted means which take into account both the beta coefficients as well as being used for comparison between means. Predicted means are evaluated by comparing the overlap between its 95% Confidence Intervals: when intervals overlap the results should be interpreted as not being significantly different at the population level. When evaluating the crude association between personality and neurocognition traits, we found that participants with higher Extraversion, Conscientiousness and Openness were significantly associated with better self-measured present memory levels whereas Neuroticism was a strong predictor of poor memory, both present and past. In addition, Extraversion and Openness were positively related with long-term retrieval. High Extraversion, Conscientiousness and Openness were associated with better cognitive performance including cognitive enjoyment and effort, while higher Neuroticism levels was negatively associated with cognitive effort. Openness was positively associated with good verbal comprehension, visual-spatial thinking, fluid reasoning, quantitative reasoning, episodic memory performance, working memory, processing speed, calculation ability and phonic/decoding skills to unfamiliar words. Individuals with higher levels of Agreeableness demonstrated negative associations with visual-spatial thinking, verbal analogical reasoning using lexical knowledge, quantitative reasoning, and calculation ability (Table **2**).

The following table adjusts previous results for the list of confounding variables, values having the same interpretation as the previous table. When evaluating the adjusted analysis, the same statistically significant associations were still present, but with slightly different predicted means (Table **3**).

When conducting the same analyses with imputed values for purposes of sensitivity analysis, all significant associations remained stable, therefore validating the robustness of our results.

## 4 DISCUSSION

To the best of our knowledge, no previous studies have evaluated the association between personality traits and neurocognitive functioning in a sample that mirrors a nationally-representative sample of elderly individuals in the United States. We found that Extraversion, Conscientiousness and Openness were positively associated with increased neurocognitive function and self-rated present memory. Extraversion and Openness also had a positive association with long-term retrieval. Agreeableness was negatively associated with several neurocognitive functions, while Neuroticism was negatively associated with memory and cognitive effort.

Personality traits have been demonstrated to predict important life outcomes such as health and longevity, educational and occupational attainment, and need for cognition [43, 44]. Previous studies comparing cognition in subjects presenting different personality types reported cognitive inhibition to be a key mechanism in the regulation of emotions [45] and personality. Specifically, higher levels of neuroticism and various forms of neurocognitive impairment predict an increased reliance on passive strategies and reduced reliance on active problem solving [46]. Higher levels of extroversion were related to greater social support seeking. In another study, intelligence, defined as one's ability to reason and to solve problems [47] was a major predictor of need for cognition and scholastic achievement [48]. This indicates a positive association between intelligence and need for cognition [47], pointing to need for cognition being a dynamic factor that changes progressively with age-related cognitive processing [49]. In relation to the association between gender, personality and cognition, previous research reported that women are more emotionally responsive than men, resulting in girls being significantly higher than boys on pro-social behavior levels [50, 51] by primary socialization agents such as parents, teachers, and peers. This finding supports the report that women score higher on emotional aspects of personal distress, empathy, and sympathy for others, all traits of agreeableness [51].

Our findings support that personality traits contribute to neurocognitive function among the elderly. Personality traits influence how an individual thinks, feels and behaves, influencing their social adjustment and life experiences. Therefore, the impact of personality traits in cognitive function likely involves interplay between various direct and indirect mechanisms [52]. These mechanisms impact not only cognitive functioning in a life-span, but also cognitive aging and its compensatory mechanisms [6, 22]. As described below, some of our results contradict previous literature. These differences likely occur because of our particularly large sample size, and also because our database included a broad neurocognitive evaluation.

Neuroticism has been consistently associated with poor cognitive function and a marked cognitive decline over one's lifetime [13, 22]. These associations have a number of possible mechanisms. First, individuals scoring high in neuroticism are prone to distraction, state anxiety and de-motivation due to worry-related thoughts [53, 54]. Second, neuroticism poses a chronic tendency to psychological stress. Chronic stress causes hyperactivation in the sympathetic nervous system and in the hypothalamic-pituitary-adrenal axis, ultimately leading to neurodenegeration [55, 56]. Third, neuroticism is a risk factor for psychiatric and physical conditions which lead toward cognitive impairment, including depression, anxiety, substance abuse, and cardiovascular disease [57-62]. Finally, neuroticism predicts poor social support and socioeconomic deprivation, ultimately resulting in reduced exposure to cognitively-stimulating activities. Differences across studies may be partly attributable to differences in either the cognitive battery or the type of personality questionnaire used. For Neuroticism, for instance, some scales may emphasize the anxiety and depression facets versus impulsivity and anger. Thus, different facets of Neuroticism may be differentially associated with cognitive function [63].

The association between Openness and higher cognitive function is also not unexpected. Openness reflects a predisposition to intellectual receptivity and flexibility in processing new information [64-66]. This personality trait also seems to have a protective effect in relation to cognitive function, occurring through an increased engagement in cognitively-stimulating activities [64], ultimately contributing toward greater cognitive reserve [67]. Individuals with high levels of cognitive reserve have an efficient cognitive network, which may help individuals to better cope with age-related changes. This also suggests that older adults engaging in highly cognitively enriching activities may have greater cognitive ability than less active older adults [68]. Openness is likely to be linked to dopaminergic function, as the neurotransmitter dopamine is one of the main drivers regarding the motivation to explore, ultimately influencing cognitive processes [69].

Conscientiousness also had a positive impact in cognitive function in our sample, likely due to its association with a series of positive behaviors including discipline, organization, and punctuality [70]. Conscientious individuals also engage less frequently in unhealthy behaviors [71], also being positively related to better cognitive functioning during their elderly years. This is likely explained by conscientiousness influencing health behaviors such as physical exercises, which leads to a larger volume in the prefrontal cortex and reduces age-related atrophy in the frontal, parietal, and temporal cortices [72, 73]. Second, individuals with better preserved cognitive functioning are likely to maintain their previous levels of Conscientiousness as they age [74]. Counter-intuitively, some authors reported a negative correlation between Conscientiousness and fluid intelligence. These authors have hypothesized that less intelligent individuals might try to compensate for lower intelligence levels through hard work [75]. Perhaps our results differ from these since the positive effect of Extraversion is more evident among the elderly, a group that is at a higher risk of social isolation.

The relationship between Extraversion and cognitive function has been controversial. Extraversion likely leads to cognitive benefits through increased social stimulation. Extrovert individuals also have a more active reward system, which might increase their cognitive efficiency through positive reinforcement. Conversely, Extraversion was previously associated with worse cognitive functioning, this association allegedly happening since extroverts have lower attention ability and decreased capacity of introspective analysis. Finally, our results were also in disagreement with reports of a positive association between Agreeableness and cognitive function [22]. We believe that a more oppositional nature might reflect a deeper intellectual activity and are more independent [76, 77]. Another possible explanation is that individuals with higher cognitive abilities do not need to develop high Agreeableness to achieve their personal goals, but they use their intellect for regulating and controlling their affective lives [78].

Despite its contribution, our study does have important limitations. Despite our best efforts in controlling for missing rates, some of our variables had particularly high rates. This limitation was minimized by using imputation algorithms followed by sensitivity analyses to ensure that our final conclusions were valid under different assumptions. Secondly, our analysis did not include longitudinal measures of cognitive function and personality. However, we have made inferences for population estimates which were not present in previous studies. Thirdly, our study is observational and analyzed associations rather than causal models. Therefore, our results should be interpreted with caution and in light or other experimental or causal models. Fourthly, since our database is cross-sectional, one cannot discard the possibility of reverse causality since we did not demonstrate that the cause preceded the outcomes. Fifthly, our study makes a number of comparisons, which could have induced us into a type I statistical error. However, our design includes a number of factors that reduced the likelihood of this issue. Specifically: (a) each of our hypotheses were based on previous literature rather than being data-driven; (b) we conducted a sensitivity analysis with adjustment for multiple testing, which led to similar conclusions; and (c) we made use of confidence intervals rather than relying exclusively on p values. Finally, it would have been important to evaluate the association with measures of mood. Unfortunately, these measures are not present in the database.

In conclusion, our results demonstrate that Extraversion, Conscientiousness and Openness personality traits are associated with good cognitive health. Individuals scoring high in Neuroticism and Agreeableness might benefit from tailored cognitive interventions to prevent age-related cognitive decline. Our results are significant in that personality traits might be used as risk factors for the future development of cognitive modifications. In this scenario, clinical and policy measures could potentially target at risk groups through prevention and early intervention programs involving the promotion of positive health behaviors and engagement in cognitively stimulating activities. There is a need of future longitudinal studies in developing a better understanding of the correlated changes in personality and cognitive ability in older adults.

## Figures and Tables

**Table 1:** Subjects socio-demographic characteristics stratified by higher than median levels of each of the personality type, with frequencies indicating values in the target population.

Variable	Extraversion ≥ 59.38 (111,251,228)	Agreeableness ≥ 83.33 (133,156,217)	Conscientiousness ≥ 80.56 (104,465,732)	Neuroticism ≥ 34.38 (132,358,975)	Openness ≥ 70 (110,070,696)
Female	64,989,565 (58.4%±4.4%)	85,296,923 (64.1%±4%)*	60,602,991 (58%±4.5%)	74,665,971 (56.4%±3.9%)	56,352,586 (51.2%±3.7%)
Race					
-White	99,738,384 (89.7%±5.3%)	119,214,754 (89.8%±4.4%)	94,655,924 (90.8%±5.2%)*	120,757,436 (91.5%±4.9%)	99,064,031 (90.3%±4.9%)*
-Black	6,742,634 (6.1%±1.7%)	9,287,486 (7%±1.6%)	7,922,116 (7.6%±1.8%)*	6,058,427 (4.6%±0.9%)	5,087,561 (4.6%±1%)*
-Other	4,770,211 (4.3%±1.4%)	4,237,798 (3.2%±1%)	1,685,100 (1.6%±0.5%)*	5,226,477 (4%±1.3%)	5,561,930 (5.1%±1.7%)*
High School degree	103,993,485 (93.5%±5.3%)	121,919,433 (91.6%±4.3%)	97,048,511 (92.9%±5.2%)	121,835,952 (92%±4.9%)	104,713,644 (95.1%±5%)*
College degree	40,688,435 (36.6%±2.4%)*	39,461,047 (29.6%±1.8%)*	33,615,922 (32.2%±2.1%)	38,097,932 (28.8%±2%)*	48,035,035 (43.6%±2.4%)*
Age (yrs)	64.94 (±0.64)	66.14 (±0.58)*	65.79 (±0.62)	64.56 (±0.56)	63.83 (±0.53)*
Marital status					
-Married	73,875,672 (66.5%±4.6%)	81,218,360 (61%±3.9%)	67,613,028 (64.7%±4.4%)	86,599,224 (65.4%±4.2%)	69,144,400 (62.9%±3.8%)
-Separated	952,722.1 (0.9%±0.3%)	1,457,399 (1.1%±0.4%)	1,207,613 (1.2%±0.5%)	872,971.3 (0.7%±0.3%)	1,090,229 (1%±0.4%)
-Divorced	15,846,024 (14.3%±2.6%)	19,895,707 (15%±2%)	14,130,846 (13.5%±2.3%)	18,314,930 (13.8%±2.4%)	18,281,502 (16.6%±2.6%)
-Widowed	17,491,954 (15.7%±2.6%)	26,903,055 (20.2%±2.4%)	17,127,904 (16.4%±2.7%)	21,196,732 (16%±2.2%)	17,170,964 (15.6%±2.9%)
-Never married	2,983,179 (2.7%±0.7%)	3,580,017 (2.7%±0.6%)	4,386,341 (4.2%±0.9%)	5,375,117 (4.1%±0.8%)	4,281,922 (3.9%±0.6%)
Area status					
-Urban	45,589,260 (41%±2.5%)	53,376,374 (40.1%±2.2%)	44,278,899 (42.4%±2.7%)	49,447,182 (37.4%±2.2%)*	47,883,743 (43.5%±2.3%)
-Suburban	21,826,702 (19.6%±2.2%)	30,367,836 (22.8%±2.2%)	22,630,215 (21.7%±2.4%)	25,022,467 (18.9%±2%)*	22,975,478 (20.9%±2.3%)
-Ex-urban	43,835,266 (39.4%±5%)	49,412,007 (37.1%±4.1%)	37,556,619 (36%±4.5%)	57,889,326 (43.7%±4.5%)*	39,211,475 (35.6%±4.5%)

footnotes: * - significantly associated variables with p value < 0.05

**Table 2:** Crude comparison of personality traits across neurocognitive constructs.

Neurocognitive variables	Extraversion ≥ 59.38	Agreeableness ≥ 83.33	Conscientiousness ≥ 80.56	Neuroticism ≥ 34.38	Openness ≥ 70
Memory	48.58 (46.62, 50.54)*	51.27 (49.14, 53.41)	49.4 (47.26, 51.54)*	54.69 (52.34, 57.05)*	49.78 (47.2, 52.36)*
Past memory	71.02 (68.81, 73.22)	72.55 (70.84, 74.26)	71.27 (69.02, 73.53)	73.49 (71.46, 75.51)*	70.89 (68.78, 73)
Episodic memory	55.64 (53.89, 57.39)	54.97 (53.47, 56.48)	54.9 (53.28, 56.52)	54.93 (53.3, 56.57)	57.67 (56.04, 59.3)*
Similarities	53.85 (52.53, 55.16)	53.51 (52.41, 54.62)	53.32 (52.09, 54.55)	52.58 (51.32, 53.84)	55.75 (54.51, 57)*
Vocabulary	54.38 (52.75, 56.01)	53.42 (51.96, 54.88)	53.55 (51.89, 55.22)	53.16 (51.78, 54.54)	57.32 (56.05, 58.59)*
Matrix reasoning	54.75 (53.33, 56.17)	53.73 (52.47, 55)	54.42 (53.01, 55.83)	54.85 (53.56, 56.14)	57.43 (56.32, 58.53)*
Block design	49.41 (48.21, 50.61)	48.95 (47.9, 50)*	50.09 (48.99, 51.2)	50.27 (49.07, 51.47)	52.3 (51.27, 53.34)*
Auditory working memory	516.99 (514.79, 519.18)	515.24 (513.08, 517.4)	515.8 (513.47, 518.13)	516.29 (514.05, 518.53)	518.86 (516.59, 521.13)*
Calculation	527.3 (525.39, 529.21)	525.47 (523.65, 527.3)*	526.96 (525.04, 528.87)	525.6 (523.51, 527.68)	530.46 (528.43, 532.49)*
Concept formation	507.93 (504.44, 511.42)	505.49 (502.72, 508.27)	507.94 (504.27, 511.61)	507.59 (504.47, 510.7)	511.22 (507.88, 514.56)*
Number series	517.53 (514.88, 520.18)	514.55 (512.24, 516.87)*	516.25 (513.72, 518.78)	515.37 (512.71, 518.02)	521.66 (518.91, 524.42)*
Picture vocabulary	557.42 (553.18, 561.67)	556.71 (553.65, 559.77)	555.44 (551.82, 559.06)	556.89 (553.54, 560.23)	564.2 (561.85, 566.56)*
Retrieval fluency	26.71 (25.54, 27.89)*	25.39 (24.56, 26.22)	25.79 (24.81, 26.77)	24.72 (23.73, 25.72)	27.45 (26.44, 28.46)*
Spatial relations	66.64 (65.53, 67.74)	65.81 (64.75, 66.86)	66.72 (65.63, 67.82)	66.39 (65.23, 67.55)	68.98 (68.04, 69.91)*
Verbal analogies	508.71 (505.22, 512.21)	506.18 (503.39, 508.96)*	508.04 (505.34, 510.73)	509.34 (506.05, 512.63)	515.19 (511.92, 518.46)*
Visual matching	75.45 (74.09, 76.81)	74.67 (73.38, 75.96)	75.44 (74.06, 76.82)	73.9 (72.55, 75.25)	75.61 (74.21, 77)*
Word attack	527.27 (523.55, 530.98)	526.65 (523.11, 530.19)	527.52 (523.59, 531.44)	526.31 (522.56, 530.06)	531.1 (527.38, 534.83)*
Cognitive enjoyment	58.04 (54.89, 61.19)*	53.98 (51.45, 56.52)	56.63 (53.45, 59.8)*	53.9 (51.49, 56.3)	65.88 (63.54, 68.22)*
Cognitive effort	67.05 (64.12, 69.97)*	63.38 (60.75, 66)	66.74 (63.69, 69.79)*	59.55 (56.79, 62.3)*	73.19 (70.58, 75.81)*
Switching	97.68 (96.92, 98.44)	96.88 (96.01, 97.76)	96.62 (95.57, 97.66)	96.66 (95.74, 97.59)	97.93 (97.23, 98.63)
Vigilance test	50.61 (49.05, 52.18)	50.45 (49.13, 51.76)	50.02 (48.64, 51.39)	50.16 (48.57, 51.76)	51.1 (49.78, 52.43)

footnotes: * - significantly associated variables with p value < 0.05

**Table 3:** Adjusted comparison of personality traits across neurocognitive constructs.

Neurocognitive variables	Extraversion ≥ 59.38	Agreeableness ≥ 83.33	Conscientiousness ≥ 80.56	Neuroticism ≥ 34.38	Openness ≥ 70
Memory	56.25 (50.68, 61.83)*	58.74 (53.25, 64.22)	57.02 (51.56, 62.47)*	61.75 (56.34, 67.17)*	58.16 (52.49, 63.83)*
Past memory	73.92 (69.58, 78.26)	75.48 (71.29, 79.67)	73.91 (69.65, 78.17)	76.32 (72.02, 80.63)*	73.87 (69.62, 78.13)
Episodic memory	51.16 (47.54, 54.78)	50.97 (47.53, 54.42)	51.14 (47.67, 54.62)	50.48 (46.9, 54.05)	52.27 (48.8, 55.74)*
Similarities	48.8 (45.71, 51.88)	49.15 (46.09, 52.21)	48.68 (45.61, 51.75)	47.89 (44.98, 50.81)	49.74 (46.73, 52.76)*
Vocabulary	49.22 (45.71, 52.74)	49.02 (45.73, 52.31)	48.85 (45.39, 52.31)	48.58 (45.61, 51.56)	51.2 (48.12, 54.28)*
Matrix reasoning	49.5 (46.88, 52.12)	49.5 (46.98, 52.02)	49.9 (47.24, 52.57)	49.54 (47.11, 51.96)	50.92 (48.45, 53.38)*
Block design	45.48 (43.23, 47.74)	45.92 (43.71, 48.14)*	46.75 (44.5, 49)	46.11 (43.92, 48.31)	47.22 (45.04, 49.41)*
Auditory working memory	509.65 (504.84, 514.46)	509.05 (504.33, 513.77)	509.39 (504.55, 514.23)	508.73 (503.86, 513.61)	510.11 (505.13, 515.09)*
Calculation	517.95 (514.27, 521.62)	518.02 (514.41, 521.64)*	518.73 (515.06, 522.4)	517.03 (513.57, 520.49)	518.9 (515.16, 522.63)*
Concept formation	498.45 (492.92, 503.98)	497.89 (492.56, 503.22)	499.79 (494.25, 505.32)	497.44 (492.09, 502.78)	499.21 (493.78, 504.64)*
Number series	503.98 (498.61, 509.34)	503.37 (498.17, 508.58)*	504.16 (498.81, 509.52)	502.13 (497.02, 507.24)	505.21 (499.71, 510.71)*
Picture vocabulary	544.52 (537.69, 551.34)	545.22 (538.76, 551.68)	543.57 (536.92, 550.22)	544.31 (538.19, 550.42)	549.74 (543.79, 555.7)*
Retrieval fluency	24.83 (22.67, 26.98)*	23.88 (21.85, 25.91)	24.22 (22.1, 26.34)	22.81 (20.83, 24.78)	24.89 (22.87, 26.9)*
Spatial relations	62.43 (59.57, 65.3)	62.37 (59.44, 65.3)	63.06 (60.09, 66.03)	61.89 (59.01, 64.77)	63.9 (61.13, 66.67)*
Verbal analogies	501.42 (496.2, 506.64)	500.9 (496.22, 505.57)*	501.92 (496.81, 507.03)	502.09 (497.26, 506.93)	505.28 (500.16, 510.41)*
Visual matching	67.05 (63.9, 70.19)	66.64 (63.38, 69.9)	67.47 (64.26, 70.68)	65.38 (62.18, 68.57)	66.6 (63.38, 69.82)*
Word attack	512.98 (503.36, 522.6)	513.62 (503.93, 523.31)	514.31 (504.32, 524.3)	512.91 (502.88, 522.94)	515.44 (505.1, 525.78)*
Cognitive enjoyment	54.44 (49.33, 59.55)*	52.12 (47.29, 56.94)	54.07 (49.08, 59.05)*	51.37 (46.75, 56)	59.56 (55.48, 63.64)*
Cognitive effort	58.67 (53.07, 64.26)*	56.25 (50.53, 61.96)	59.04 (53.35, 64.73)*	51.81 (46.44, 57.19)*	62.3 (55.85, 68.75)*
Switching	97.3 (95.28, 99.32)	96.57 (94.45, 98.69)	96.28 (94.16, 98.4)	96.16 (94.02, 98.31)	97.45 (95.46, 99.43)
Vigilance test	46.22 (42.87, 49.56)	46.42 (43.13, 49.7)	45.78 (42.33, 49.23)	45.62 (42.18, 49.05)	46.02 (42.66, 49.38)

footnotes: * - significantly associated variables with p value < 0.05.
